# ﻿*Campanulaluzhijiangensis* (Campanulaceae), a new species from Yunnan, southwest China

**DOI:** 10.3897/phytokeys.206.87109

**Published:** 2022-08-26

**Authors:** Ting-Ting Wang, Zeng-Yan Dang, Feng Yang, Huan-Chong Wang

**Affiliations:** 1 School of Ecology and Environmental Science, Yunnan University, Kunming 650091, Yunnan, China; 2 School of Life Sciences, Yunnan University, Kunming 650500, Yunnan, China; 3 Herbarium of Yunnan University, Kunming 650091, Yunnan, China

**Keywords:** *Campanulamekongensis*, Campanuleae, endemism, Luzhijiang Valley, morphology, taxonomy

## Abstract

*Campanulaluzhijiangensis* (Campanulaceae: Campanuleae) is described and illustrated as a new species from Yunnan, southwest China. The new species is mainly characterized by its relatively gracile stems polyphyllous, small and oblanceolate leaves, and flowers and fruits with small size within Chinese *Campanula*. It is only known from a single locality in the valley of the Luzhijiang River, usually occurring in the rock crevices, xerophilous scrubs or grasslands. A table of morphological characters comparing the new species with its closest relatives is provided along with a key to the species of *Campanula* from Yunnan Province, as well as a preliminary conservation assessment of *C.luzhijiangensis* under the IUCN criteria.

## ﻿Introduction

The tribe Campanuleae, comprising more than 620 species, is the largest tribe in the Campanulaceae*s. str.* (excluding Cyphiaceae, Cyphocarpaceae, Lobeliaceae, and Nemacladaceae) (Lammers 2007a, b; Xu and Hong 2020). Within this tribe, the generic classification still remains contentious, especially in the delimitation of genus *Campanulas. l.*, which was found to be polyphyletic by the recent molecular phylogenetic analyses (Roquet et al. 2008; Lakušić et al. 2013; Crowl et al. 2016; Liveri et al. 2019; Xu and Hong 2020). *Campanulas. l.* consists of about 420–600 species (Lammers 2007b), most of which are perennials with alternate cauline leaves, flowers with radial flora symmetry and composed of a calyx with five persistent sepals, campanulate, tubular-campanulate, or funnelform corolla with five lobes, filaments dilated, an inferior ovary, and the capsule dehiscent at side. Members of this genus are widely distributed in temperate regions of the Northern Hemisphere (Borsch et al. 2009), and are especially abundant in the Mediterranean region and the Middle East (Fedorov and Kovanda 1978; Contandriopoulos 1984). They inhabit a wide range of habitats, including meadows, woodland-edges, moorlands, and cliffs, as well as steppe and mountainous habitats (Fedorov 1957; Kovacic 2004).

There are 22 species of *Campanula* in China, of which 11 are endemic (Wang and Hong 2000; Hong et al. 2011). During recent field investigations in Yunnan province of southwestern China, we found an unknown species of *Campanula*. After detailed comparison with morphologically similar taxa and extensive analysis of the relevant literature, it became clear it represents an undescribed species.

## ﻿Materials and methods

This study followed the normal practice of plant taxonomic survey and herbarium taxonomy. Morphological studies of the new species were based on observation of living plants and specimens from the Luzhijiang Valley in Yimen County, Yunnan Province, southwest China. Morphological features were studied under a stereomicroscope (Olympus SZX2, Tokyo, Japan), and measurements were made using a ruler or a micrometer. Digital images of type specimens of its congeners available at the JSTOR Global Plants (http://plants.jstor.org/) and the Chinese Virtual Herbarium (https://www.cvh.ac.cn/), as well as relative collections housed at CDBI, KUN, PE, PYU and YUKU (acronyms according to Thiers 2022), were examined and compared with the new species. Pertinent taxonomic literature (e.g. Hong 1983, 2015; Huang 1991; Hong et al. 2011) were extensively consulted.

## ﻿Taxonomy

### ﻿ Campanula luzhijiangensis

Taxon classificationPlantaeAsteralesCampanulaceae

Huan C. Wang & T. T. Wang sp. nov.

4694143C-BBDA-5D3B-A842-9C0E9F69FD1C

urn:lsid:ipni.org:names:77303990-1

Figs 1 , 2 , 3

#### Type.

China. Yunnan Province: Yimen County, Luzhi Town, Luzhijiang Valley, Xiaoluzhi, 24°40'53"N, 101°58'19"E, elev. 1450 m, 25 September 2021, *H. C. Wang et al. YM15319* (holotype YUKU!, isotypes YUKU!).

**Figure 1.**
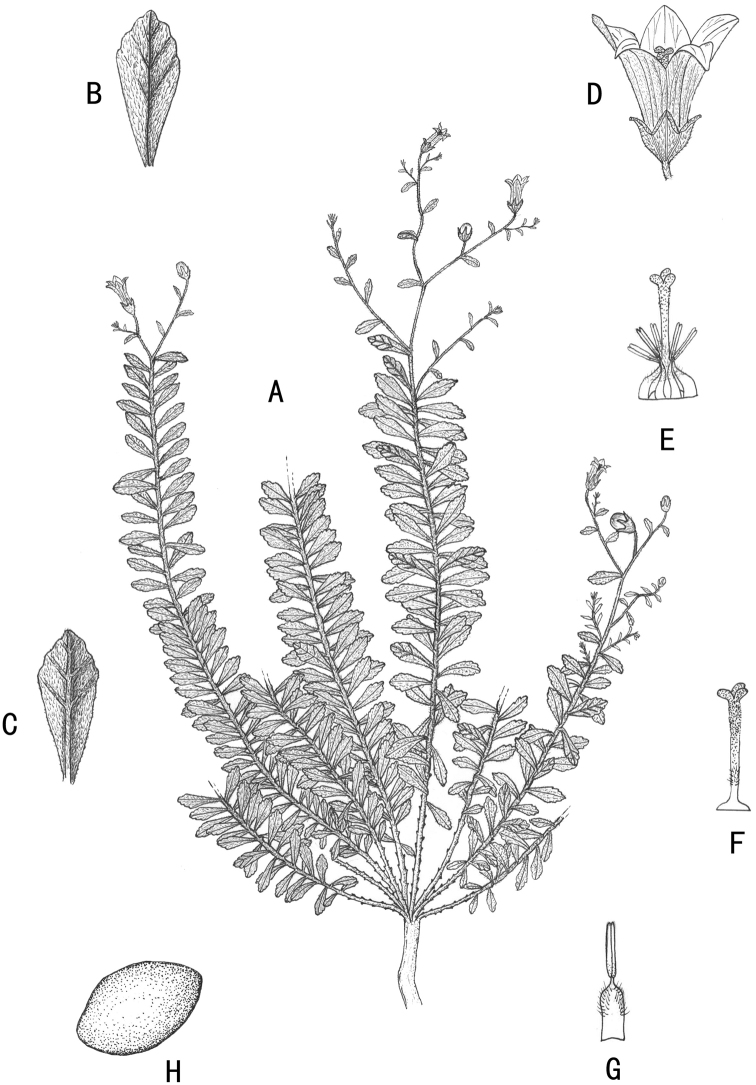
*Campanulaluzhijiangensis***A** habit **B** adaxial surface of leaf **C** abaxial surface of leaf **D** flower **E** style and stamens **F** style **G** stamen **H** seed.

#### Diagnosis.

*Campanulaluzhijiangensis* is most similar to *C.mekongensis* Diels ex C. Y. Wu, but clearly distinguished from the latter by its stems with numerous leaf scars at base, leaves usually oblanceolate, relatively small, 0.3–2.0 cm long, 0.1–0.3 (– 0.5) cm wide, margin subentire or sparsely crenate, slightly recurved, hypanthium densely villous throughout, calyx lobes usually ovate, 1–2 mm long, 1.0–1.5 mm wide, corolla tubular-campanulate, tube 2–4 mm in diameter. In contrast, *C.mekongensis* has stems without leaf scars at base, leaves oblong, narrowly obovate or oblanceolate, 0.5–3.0 cm long, 0.3–1.2 cm wide, margin not recurved, serrate, hypanthium only hispid along ribs, calyx lobes subulate, 2–4 mm long, less than 1 mm wide, corolla campanulate, tube 6–10 mm in diameter.

**Figure 2.**
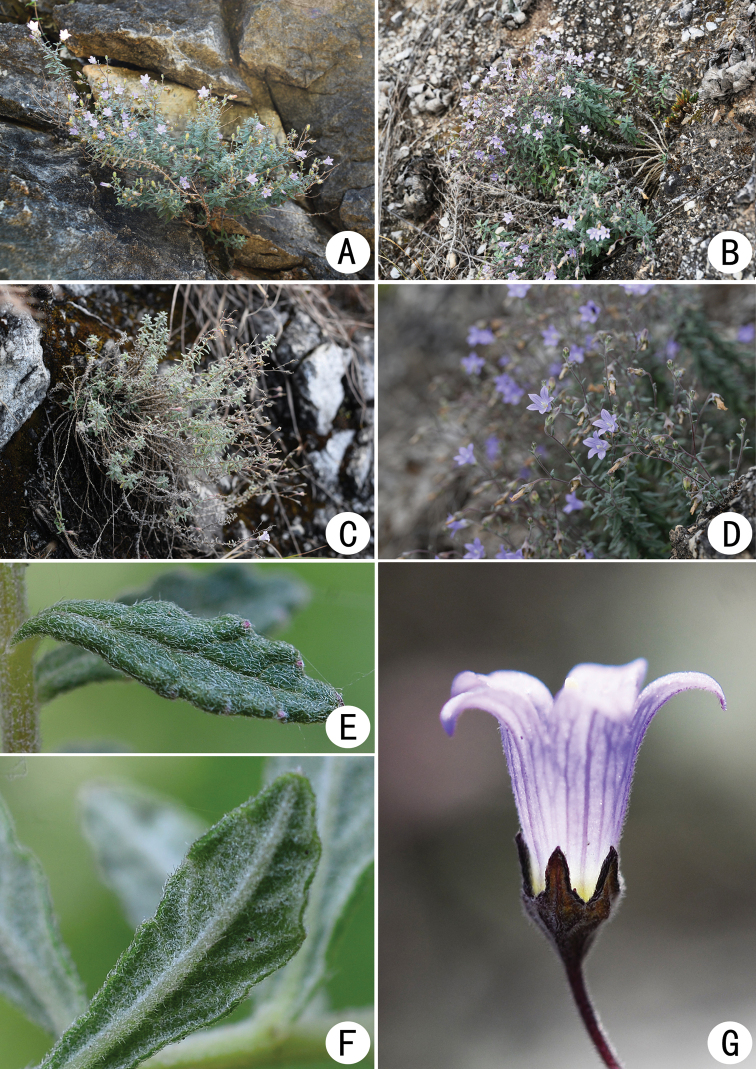
*Campanulaluzhijiangensis***A, B** habit **C** plants in fruiting stage **D** plants in flowering stage **E** adaxial surface of leaf **F** abaxial surface of leaf **G** flower (side view).

#### Description.

Herbs perennial, caespitose. Rootstock woody, naked, with numerous ascending stems. Stems polyphyllous, usually simple at base, rarely branched, slightly lignified, purplish, densely white villous, 10–30 cm long. Leaves alternate, sessile or subsessile, basal leaves withering or caducous; blades usually oblanceolate, rarely elliptic, 0.3–2.0 cm long, 0.1–0.3 (–0.5) cm wide, abaxially densely villous, adaxially appressed pubescent, apex obtuse to acute, margin subentire or sparsely crenate, slightly recurved, base cuneate. Inflorescences terminal, thyrsiform; rachis and branches gracile, indumentum similar to that of the stems; bracts oblanceolate, lanceolate to linear, 0.1–0.2 cm long, 0.7–0.9 mm wide. Flowers erect or ascending, rarely reflexed; pedicels gracile, villous, 0.5–1.0 cm long, 0.2–0.3 mm in diameter; hypanthium obconic, longitudinally ribbed, densely spreading villous, base cuneate, calyx lobes ovate, 1–2 mm long, 1.0–1.5 mm wide at base, acute to acuminate at apex, margin slightly reflexed, serrulate. Corolla blue, blue-white or lilac, tubular-campanulate, 5–10 mm long, externally pubescent, internally glabrous; tube subconic, 4–8 mm long, 2–4 mm in diameter; corolla lobes ovate to ovate-lanceolate, or nearly oblong, 2.5–7.0 mm long, acute at apex. Stamens 5, included, shorter than style; filaments ca. 3 mm long, base dilated into flakes, dilated part nearly elliptic, densely villose, connivent around the style at the anthesis; anthers clavate, ca. 2 mm long, light yellow. Style slightly exserted, ca. 6 mm long, base glabrous, middle and lower part with hairs, upper part papillose; stigma 3-fid, 1.0–1.3 mm long. Capsule obconic, 3-poricidal toward base, apical calyx lobes persistent. Seed elliptic, shiny, 0.4–0.5 mm long, 0.2–0.3 mm wide.

**Figure 3.**
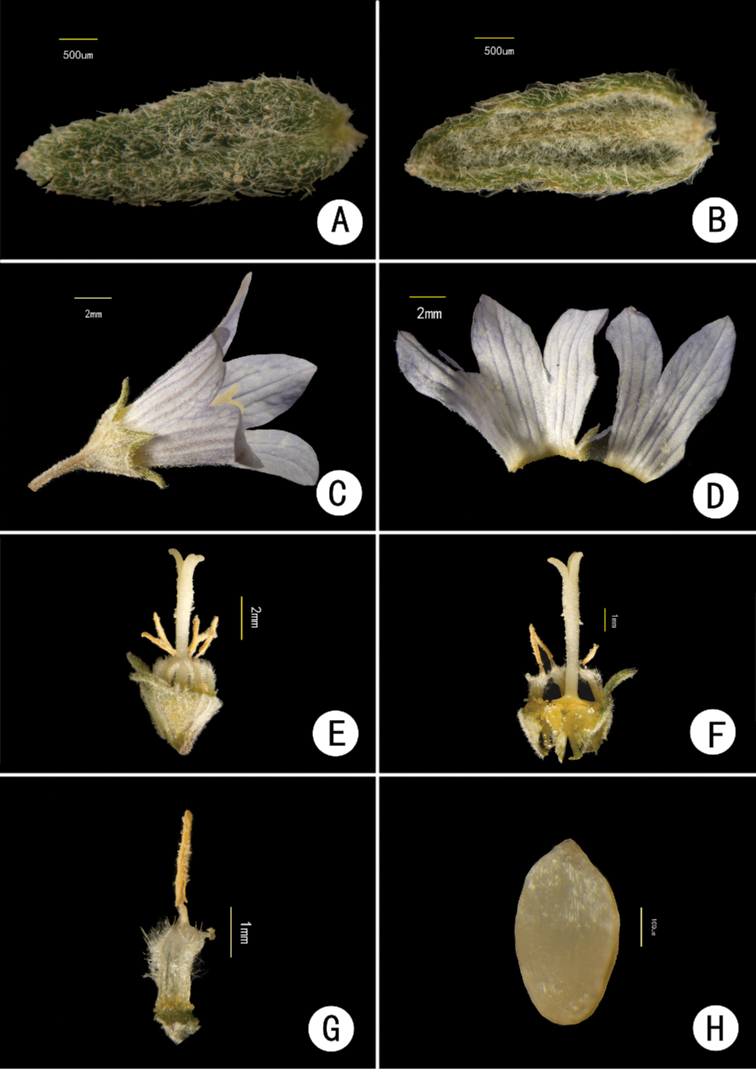
*Campanulaluzhijiangensis***A** adaxial surface of bract **B** abaxial surface of bract **C** flower (side view) **D** corolla dissected **E** stamens and pistil **F** style, stamens and dissected hypanthium **G** stamen **H** seed.

#### Phenology.

*Campanulaluzhijiangensis* has a relatively long flowering period; it usually flowers from August to January of the following year, and fruits from September to February.

#### Etymology.

The specific epithet *luzhijiangensis* is derived from the type locality of the new species, the Luzhijiang Valley, and the Latin suffix *ensis*, indicating the place of origin or growth.

#### Habitat and distribution.

**Figure 4.**
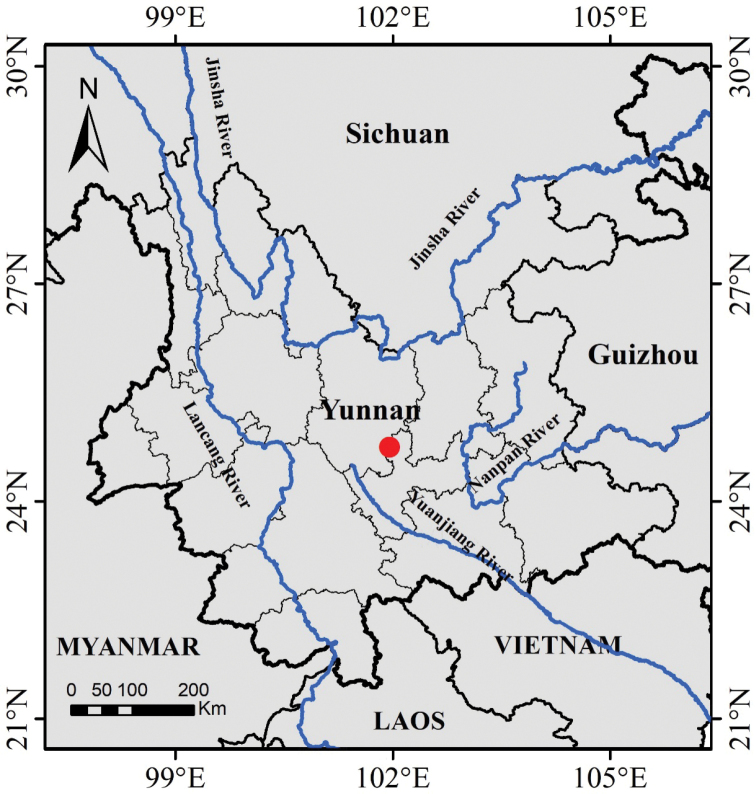
Geographical distribution of *Campanulaluzhijiangensis* (red dot).

*Campanulaluzhijiangensis* appears to be a rare species endemic to Yunnan, southwest China. It is only known from the type locality in the valley of the Luzhijiang River, an upper tributary of the Hong (Red) River that flows from Yunnan in southwest China through northern Vietnam to the Gulf of Tonkin (Fig. 4). The climate in Luzhijiang Valley is semi-dry and hot, with an annual average temperature of 21.0 °C and a total annual precipitation of 822.8 mm. *Campanulaluzhijiangensis* usually occurs in the rock crevices, xerophilous scrubs or grasslands between 1250 and 1500 m elevation. Associated vegetation includes *Phyllanthusemblica* Linn. (Phyllanthaceae), *Paliurusorientalis* (Franch.) Hemsl. (Rhamnaceae), *Dalbergiayunnanensis* Franch. (Fabaceae), *Symphoricarpossinensis* Rehd. (Caprifoliaceae), *Duhaldealachnocephala* Huan C. Wang & Feng Yang (Asteraceae) (an endemic species described by Yang et al. (2022)), *Pterygiellaluzhijiangensis* Huan C. Wang (Orobanchaceae), *Sileneotodonta* Franch. (Caryophyllaceae), *Spodiopogonsagittifolius* Rendle (Poaceae), *Heteropogoncontortus* (Linn.) Beauv. ex Roem. et Schult. (Poaceae) and *Themedacaudata* (Nees ex Hooker et Arnott) A. Camus (Poaceae).

#### Conservation status.

*Campanulaluzhijiangensis* is a rare species with a restricted distribution and small population size. Currently, it is only known from a single locality in the Luzhijiang River Valley in the Yimen County, southwest China, where the estimated area of occupancy (AOO) is less than 20 km^2^. The total population size is estimated to be fewer than 500 mature individuals. Following the IUCN guidelines (IUCN 2012, 2022), this new species should be classified as Vulnerable [VU (D1, D2)].

## ﻿Discussion

On the basis of living and herbarium materials, *Campanulaluzhijiangensis* is well differentiated from the other species of the *Campanula* found in southwest China and the adjacent regions in several and significant features regarding the vegetative and reproductive structure. In particular, it is characterized by its relatively gracile stems polyphyllous, small and oblanceolate leaves, small flowers and fruits. Morphologically, *C.luzhijiangensis* is most similar to *C.mekongensis*, an endemic found in southwest (Xishuangbanna) and northwest (Nujiang Valley) Yunnan Province, southwest China (Hong 2015), and it is somewhat close to *C.cana* Wall. in sharing similar indumentum, and to *C.pallida* Wall., a variable and common species also found in southwest China, in inflorescence structure and shape of calyx lobe. Nevertheless, there are several morphological features distinguishing *Campanulaluzhijiangensis* from the other three species (see Table 1). Species of *Campanula* found in Yunnan Province, southwest China, can be distinguished through the morphological characters presented in the following identification key modified from Hong (1983, 2015) and Hong et al. (2011).

**Table 1.** Morphological comparison of *Campanulaluzhijiangensis*, *C.mekongensis*, *C.cana* and *C.pallida*.

Characters	*C.luzhijiangensis*	*C.mekongensis*	*C.cana*	*C.pallida*
Stem length (cm)	10–30	20–30	15–30	20–50 (–60)
Stem indumentum	densely white villous	pilose to sparely villous	densely white villous, sometimes tomentose	hirsute to hispid
Leaf scars at lower part of stem	numerous	absent	absent or few	absent or few
Leaf shape	oblanceolate	oblong, narrowly obovate or oblanceolate	ovate, elliptic, oblanceolate or linear-lanceolate	elliptic, rhombic-elliptic or oblong
Leaf size (cm)	0.3–2.0 × 0.1–0.3 (–0.5)	0.5–3.0 × 0.3–1.2	0.4–2.5 × 0.3–1.0	1.0–4.0 × 0.3–1.5
Leaf apex	obtuse to acute	usually acute, rarely obtuse	obtuse to acute	acute to acuminate
Leaf margin	subentire or sparsely crenate, slightly recurved	serrate, not recurved	subentire, crenulate, or serrate, slightly recurved	denticulate or almost entire, not recurved
Flower	erect or ascending, rarely reflexed	ascending to nodding	erect or ascending, sometimes pendent	usually pendent
Length of flower pedicel (cm)	0.5–1.0	up to 2.0	0.5–3.0	0.4–2.0
Hypanthium	obconic, externally spreading villous	obconic or campanulate, only hispid along ribs	obdeltoid to broadly obconical, externally villous to tomentose	obconic or campanulate, externally hirsute to hispid
Shape of calyx lobes	ovate	subulate	subulate or narrowly triangular	deltoid, narrowly triangular, or subulate
Size of calyx lobes (mm)	1–2 × 1.0–1.5	2–4 mm long, less than 1 mm wide	3–5 × 2–3	2–8 × 2–5
Length of corolla (mm)	5–10	8	10–15	4–15
Diameter of corolla tube (mm)	2–4	6–10	2–7	5–10

### ﻿Identification key to the species of *Campanula* found in Yunnan Province, China

**Table:** 

1	Capsule poricidal toward base; stems with numerous flowers, solitary or in various types of inflorescences; leaves many and evenly distributed on stems; basal leaves usually wilted at anthesis	**2**
–	Capsule poricidal above middle; flowers solitary, terminal, or several terminal on main stems and branches; cauline leaves mostly toward base, upper cauline leaves sessile or nearly so, usually linear if present; basal leaves persistent at anthesis	**8**
2	Annual herbs; rosulate basal leaves sometimes present at anthesis	***C.dimorphantha***
–	Perennial herbs; basal leaves often absent at anthesis	**3**
3	Stems with numerous leaf scars at base; flowers small, calyx lobes 1–2 mm long, corolla 5–10 mm long	***C.luzhijiangensis* sp. nov.**
–	Stems with few or without leaf scars at base; flowers relatively large, calyx lobes 2–10 mm long, corollas more than 10 mm long	**4**
4	Calyx tube hairy only along veins; lateral branches with several flowers; calyx lobes subulate, sinus between lobes truncate-obtuse	***C.mekongensis***
–	Calyx tube densely hairy; lateral branches with a solitary flower or single simple inflorescence; calyx lobes subulate-triangular to deltoid, overlapping, or sinus acute	**5**
5	Calyx lobes deltoid, with a pair of large teeth; stems long and prostrate; cauline leaves of lower half of stem wilted at anthesis, rest of leaves pannose abaxially, sessile, suborbicular	***C.yunnanensis***
–	Calyx lobes subulate-triangular, rarely subdeltoid, with or without teeth; stems erect or diffuse; lower cauline leaves often present at anthesis; leaves sparsely hispid or densely pannose abaxially, elliptic, rhombic, or linear-elliptic	**6**
6	Style strongly exserted; anthers completely or partially connivent	***C.chinensis***
–	Style included; anthers completely free	**7**
7	Calyx lobes narrowly triangular to subdeltoid, toothed or not; leaves often hispid, less frequently pannose abaxially; stems single or several from one caudex, erect or ascending	***C.pallida***
–	Calyx lobes subulate-triangular to narrowly triangular, rarely toothed; leaves densely pannose abaxially; stems usually numerous from one caudex, often diffuse, less often ascending	***C.cana***
8	Plants with horizontal rhizomes; stems simple; stems and leaves glabrous	**9**
–	Plants without horizontal rhizomes; stems simple or branched; stems and leaves variously pubescent	**10**
9	Hypanthium narrowly cylindrical; calyx lobes filiform, longer than corolla	***C.aristata***
–	Hypanthium obovoid or obconic; calyx lobes subulate or narrowly triangular, shorter than corolla	***C.modesta***
10	Flowers pendent	**11**
–	Flowers erect	**12**
11	Plants 20–50 cm tall; calyx lobes 1–5.5 mm; corolla lobes as long as tube	***C.delavayi***
–	Plants 6–33 cm tall; calyx lobes 3–12 mm; corolla lobes ca. 1/2 as long as tube	***C.crenulata***
12	Basal leaves cordate-reniform; stems slender but not filiform, hairy, lower half with cordate to ovate-lanceolate leaves; capsule 4–8 mm	***C.calcicola***
–	Basal leaves cordate; stems filiform, glabrous or subglabrous, with mostly linear leaves; capsule 9–19 mm	***C.chrysospleniifolia***

**Additional specimens examined (*paratypes*): China. Yunnan**: Yimen County, Luzhi Town, Xiaoluzhi, 20 October 1965, *W. M. Zhu et al. 1375* (YUKU); ibid., 9 August 2016, *H. C. Wang et al. YM1052* (YUKU); ibid., 3 October 2016, *H. C. Wang et al. YM1270* (YUKU); ibid., 18 January 2018, *H. C. Wang et al. YM8028* (YUKU); ibid., 12 November 2019, *H. C. Wang et al. YM8304, YM8327* (YUKU).

## Supplementary Material

XML Treatment for Campanula luzhijiangensis

## References

[B1] Borsch T Korotkova N Raus T Lobin W Löhne C (2009) The *pet*D group II intron as a species level marker: Utility for tree inference and species identification in the diverse genus *Campanula* (Campanulaceae). Willdenowia 39(1): 7–33. 10.3372/wi.39.39101

[B2] Contandriopoulos J (1984) Differentiation and evolution of the genus *Campanula* in the Mediterranean region. In: Grant WF (Ed.) Plant biosystematics.Academic Press, Toronto, 141–158. 10.1016/B978-0-12-295680-5.50014-7

[B3] Crowl AA Miles NW Visger CJ Hansen K Ayers T Haberle R Cellinese N (2016) A global perspective on Campanulaceae: Biogeographic, genomic, and floral evolution. American Journal of Botany 103(2): 233–245. 10.3732/ajb.1500450 26865121

[B4] Fedorov AA (1957) Campanulaceae. In: Schischkin BK, Bobrov EG (Eds) Flora of the USSR, vol. 24. Academy of sciences of the USSR, Moscow 92–321.

[B5] Fedorov AA Kovanda M (1978) *Campanula* Linnaeus. In: Tutin TG (Ed.) Flora Europaea, vol.4. Cambridge University Press, Cambridge, 74–93.

[B6] Hong DY (1983) *Campanula* Linnaeus and *Adenophora* Fischer. In: Hong DY (Ed.) Flora Reipublicae Popularis Sinicae, vol.73(2). Science Press, Beijing, 78–92.

[B7] Hong DY (2015) *Campanula* Linnaeus. In: Hong DY (Ed.) Flora of Pan-Himalaya, vol. 47. Science Press, Beijing, 292 pp.

[B8] Hong DY Lammers TG Klein LL (2011) *Campanula* Linnaeus. In: Wu ZY Raven PH (Eds) Flora of China, vol.19. Science Press, Beijing & Missouri Botanical Garden Press, St. Louis, 530–536.

[B9] Huang SH (1991) Campanulaceae. In: Wu ZY (Ed.) Flora Yunnanica, vol.5. Science Press, Beijing, 452–509.

[B10] IUCN (2012) IUCN Red List Categories and Criteria. Version 3.1. (2^nd^ edn.). IUCN, Gland, Switzerland & Cambridge, 32 pp.

[B11] IUCN (2022) Guidelines for Using the IUCN Red List Categories and Criteria. Version 15. Prepared by the Standards and Petitions Committee.

[B12] Kovacic S (2004) The genus *Campanula* L. (Campanulaceae) in Croatia, circum-Adriatic and west Balkan region. Acta Botanica Croatica 63(2): 171–202.

[B13] Lakušić D Liber Z Nikolić T Surina B Kovačić S Bogdanović S Stefanović S (2013) Molecular phylogeny of *Campanulapyramidalis* species complex (Campanulaceae) inferred from chloroplast and nuclear non-coding sequences and its taxonomic implications. Taxon 62(3): 505–524. 10.12705/623.1

[B14] Lammers TG (2007a) Campanulaceae. In: Kadereit JW Jeffrey C (Eds) The Families and Genera of Vascular Plants, vol.8. Springer, Berlin, 26–56.

[B15] Lammers TG (2007b) World Checklist and Bibliography of Campanulaceae. Royal Botanic Gardens, Kew.

[B16] Liveri E Crowl AA Cellinese N (2019) Past, present, and future of *Campanula* (Campanulaceae) systematics-areview. Botanika Chronika 22: 209–222.

[B17] Roquet C Sáez L Aldasoro JJ Susanna A Alarcon ML Garcias-Jacad N (2008) Natural delineation, molecular phylogeny and floral evolution in *Campanula*. Systematic Botany 33(1): 203–217. 10.1600/036364408783887465

[B18] Thiers B (2022) [continuously updated] Index Herbariorum: a global directory of public herbaria and associated staff. New York Botanical Garden’s Virtual Herbarium. http://sweetgum.nybg.org/science/ih/ [accessed 28.05.2022]

[B19] Wang LZ Hong DY (2000) *Campanulagansuensis* (Campanulaceae), a new species from China, and its systematic position. Botanical Bulletin of Academia Sinica 41(2): 159–163.

[B20] Xu C Hong DY (2020) Phylogenetic analyses confirm polyphyly of the genus *Campanula* (Campanulaceae*s. str.*), leading to a proposal for generic reappraisal. Journal of Systematics and Evolution 59(3): 475–489. 10.1111/jse.12586

[B21] Yang F Ye JY Huang QC Wang QP Wang HC (2022) *Duhaldealachnocephala* (Asteraceae: Inuleae: Inulinae), a new species from Yunnan, southwest China. Taiwania 67(2): 217–222.

